# Metagenomic next-generation sequencing in detecting pathogens in pediatric oncology patients with suspected bloodstream infections

**DOI:** 10.1038/s41390-023-02776-y

**Published:** 2023-10-19

**Authors:** Jing Wu, Wenting Song, Hui Yan, Chengjuan Luo, Wenting Hu, Li Xie, Nan Shen, Qing Cao, Xi Mo, Kang An, Yue Tao

**Affiliations:** 1 Pediatric Translational Medicine Institute, Shanghai Children’s Medical Center, Shanghai Jiao Tong University School of Medicine, Shanghai, China; 2 Department of Infectious Diseases, Shanghai Jiao Tong University School of Medicine, Shanghai, China; 3 Department of Hematology and Oncology, Shanghai Jiao Tong University School of Medicine, Shanghai, China; 4 Clinical Research Institute, Shanghai Jiao Tong University School of Medicine, Shanghai, China

## Abstract

**Background:**

Studies on mNGS application in pediatric oncology patients, who are at high risk of infection, are quite limited.

**Methods:**

From March 2020 to June 2022, a total of 224 blood samples from 195 pediatric oncology patients who were suspected as bloodstream infections were enrolled in this study. Their clinical and laboratory data were retrospectively reviewed, and the diagnostic performance of mNGS was assessed.

**Results:**

Compared to the reference tests, mNGS showed significantly higher sensitivity (89.8% vs 32.5%, *P* < 0.001) and clinical agreement (76.3% vs 51.3%, *P* < 0.001) in detecting potential pathogens and distinguishing BSI from non-BSI. Especially, mNGS had an outstanding performance for virus detection, contributing to 100% clinical diagnosed virus. Samples from patients with neutropenia showed higher incidence of bacterial infections (*P* = 0.035). The most identified bacteria were *Escherichia coli*, and the overall infections by gram-negative bacteria were significantly more prevalent than those by gram-positive ones (90% vs 10%, *P* < 0.001). Overall, mNGS had an impact on the antimicrobial regimens’ usage in 54.3% of the samples in this study.

**Conclusions:**

mNGS has the advantage of rapid and effective pathogen diagnosis in pediatric oncology patients with suspected BSI, especially for virus.

**Impact:**

- Compared with reference tests, mNGS showed significantly higher sensitivity and clinical agreement in detecting potential pathogens and distinguishing bloodstream infections (BSI) from non-BSI.
- mNGS is particularly prominent in clinical diagnosed virus detection.
- The incidence of bacterial infection was higher in patients with neutropenia, and the overall infection rate of Gram-negative bacteria was significantly higher than that of Gram-positive bacteria.
- mNGS affects the antimicrobial regimens’ usage in more than half of patients.

## Introduction

Bloodstream infections (BSI) is a common and life-threatening complication of cancer treatment, which can lead to medical emergencies such as severe sepsis.^1^ Due to the high morbidity and mortality of BSI, it is essential to give the patients a timely pathogen detection and diagnosis to treat the infections early with appropriate antimicrobial agents.^2,3^ However, because of lacking rapid and effective diagnostic methodologies to identify causative organism, patients are usually treated with empirical antimicrobial therapy immediately after infection occurring.^4^ Consequently, inaccurate diagnosis and treatment may lead to disease progression and even death; overuse of antimicrobials leads to the increase of multi-drug resistant bacteria and decreases the diversity of patients’ gut microbiota, which adversely affect the long-term prognosis of the patients.^5^

Despite the obvious disadvantages, such as long turn-around time (TAT), low positive detection rate, and decreased sensitivity after antibiotic treatment, traditional blood culture is still the main method and gold standard to detect the causative microorganisms for BSI. New pathogen diagnosis methods with high sensitivity and short TAT are urgently needed. With the rapid development of molecular biology, nucleic acid sequencing-based diagnosis, such as metagenomic next-generation sequencing (mNGS), which only requires a small amount of blood sample, provides a useful tool to solve this problem. Compared to the traditional tests, mNGS is more sensitive, unbiased, and unaffected by antibiotics in detecting potential pathogenic microorganisms. It also showed unparalleled advantages in detecting polymicrobial infections, as well as detecting unculturable or novel pathogens.^6,7^ Therefore, mNGS is becoming a promising method to address some of the challenges in pathogen diagnosis.

Numerous reports have studied the potential application of mNGS in a variety of settings, especially in respiratory tract infection and central nervous system infection.^8,9^ However, studies on mNGS application in pediatric oncology patients, who are at high risk of infection, are still quite limited.^10^ Most of the oncology patients were immunocompromised, especially for those with hematologic malignancy, who are more prone to be infected with unusual or polymicrobial pathogens. Oncology patients may also have special clinical manifestation, including atypical clinical presentations of common pathogens, or presentation of symptoms mimic infections.^11,12^ Therefore, pathogen detection in these patients is particularly complicated and challenging. More studies are still needed to evaluate the application of mNGS in oncology patients.

Herein, we retrospectively analyzed the clinical data of 195 pediatric oncology patients who were suspected for BSI and received mNGS testing. We compared the diagnostic performance of mNGS and reference tests, and assessed the clinical impact of mNGS, aiming to help give a better understanding of the clinical application of mNGS on pediatric oncology patients.

## Materials and methods

### Study participants

One hundred and ninety-five oncology patients who were suspected as BSI and received mNGS examination from March 2020 to June 2022 at Shanghai Children’s Medical Center were included in the present study. Suspected BSI was defined as the presence of a sudden high fever (temperature ≥38.5 °C) accompanied by hemodynamic instability that could not be attributed to a localized infection at another anatomical site, as well as an increase of 2 points or more in the sepsis-related organ failure assessment (SOFA) score as previously described.^13^ The patients’ information, including demographic characteristics, clinical history, laboratory examination information, reference test results, mNGS results, and treatment, were collected from patients' medical records during their in-patient treatment period.

**Fig. 1:**
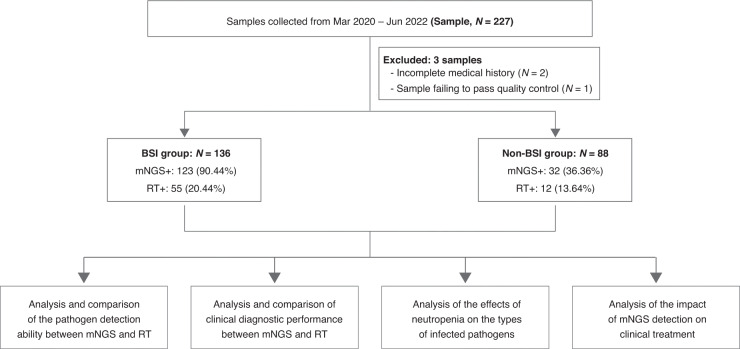
Flowchart of the present study. A total of 224 samples were included in this study after excluding 3 samples. According to the final clinical diagnosis, the 224 samples were divided into bloodstream infection (BSI) group and non-BIS group. All data was then analyzed accordingly. mNGS metagenomic next-generation sequencing, RT reference test.

A total of 227 samples were sent for mNGS and reference tests, 3 of which were excluded because of incomplete medical history or unqualified samples for mNGS, thus 224 samples were included in this study (Fig. 1). All samples were sent for mNGS and routine microbiological tests. The mNGS results for each sample included the detected microorganism and reads per million (RPM) information. BSI is defined by positive blood cultures in a patient with systemic signs of infection and may be either secondary to a documented source or primary—that is, without identified origin. Patients who had one or more pathogens identified were diagnosed as definite BSI. In certain cases, even no pathogen was detected, a diagnosis of BSI was still made if patients exhibited symptoms of infection, responded positively to anti-infection treatments, and laboratory tests provided supporting evidence.^14–16^ All the samples were divided into BSI group and non-BSI group according to the final clinical diagnosis, which was determined by retrospective, in-depth chart review conducted independently by two senior physicians with BSI expertise.

The study was approved by the Institutional Review Board and the Ethics Committee of Shanghai Children’s Medical Center (SCMCIRB-K2017070), and written informed consent was obtained from all patients and/or their parents.

### Sample collection

Blood samples were collected using sterile equipment after disinfecting the skin, then promptly transported to the laboratory. The sample handling process was conducted in a clean operating area, with surfaces disinfected. Operators wore masks, gloves, and other protective equipment to prevent any personnel contamination. Only 13.4% samples (30/224) that underwent mNGS sequencing were collected on the same day as the samples for reference tests, and 59.8% samples (134/224) that underwent mNGS sequencing were collected within 3 days following reference tests (Supplementary Table 1).

### Process of mNGS

The nucleic acid extraction, library preparation and sequencing were carried out as previous study.^17^ Briefly, approximately 3–5 mL peripheral blood was collected, and plasma was obtained by a centrifugation at 4 °C, 1600 rpm, for 10 min. Plasma DNA and RNA were extracted by using QIAamp^®^ UCP Pathogen DNA Kit and QIAamp^®^ Viral RNA Kit (Qiagen) according to the manufacturer’s instructions, respectively. To include negative controls, PBMC samples (10^5^ cells/mL) obtained from healthy donors were prepared and underwent the same protocol and processing conditions as the test sample materials. Additionally, sterile deionized water was included as a non-template control (NTC) alongside the specimens. Finally, to help to differentiate pathogenic microorganisms from background microorganisms, a comprehensive list of suspected background or contaminated microorganisms were included at the end of the reports. Nextera XT DNA Library Prep Kit (Illumina) was used to construct libraries for sequencing (Illumina Nextseq CN500).

### Bioinformatics analyses

The bioinformatics analyses were conducted as previously described.^17^ Trimmomatic was utilized to eliminate low-quality reads, adapter contamination, duplicate reads, as well as reads shorter than 50 bp. Kcomplexity was employed to remove low complexity reads. Reference genomes, were obtained from the National Center for Biotechnology Information (https://benlangmead.github.io/aws-indexes/k2), and reference databases were created using Kraken2 v2.0.8beta. Then, human sequences were identified and removed by aligning the reads to the hg38 using Burrows-Wheeler Aligner software. Taxonomic classification was conducted using Kraken2 v2.0.8beta, and mapped reads at the species level were calculated using Bracken with default parameters.

For each sample, approximately 20 million reads were generated. For pathogens with background reads in the negative control, a positive detection was reported for a given species or genus if the reads per million (RPM) ratio, or RPM-r, was ≥10. The RPM-r was calculated as the ratio of RPM_sample_ to RPM_NC_, where RPM_NC_ refers to the RPM of a given species or genus in the negative control. For pathogens without background reads in the negative control, the RPM threshold was set to ≥0.05. Besides, to reduce false positive results, we implemented a penalty system that reduced the RPM of microorganisms sharing a genus or family designation, if the species or genus appeared in non-template controls. Specifically, we used a penalty of 5% and 10% for species and genus, respectively.

### Reference tests

Reference tests refer to routine clinical microbiological assays, including bacterial and fungal culture, PCR detection for Epstein-Barr virus (EBV) and Cytomegalovirus (CMV), β-D-glucan (G) test, and galactomannan (GM) test. Bacterial and fungal cultures were identified using the BD BACTEC FX automated blood culture system (Becton, Dickinson and Company) and the VITEK MS mass spectrometer (Bio-Meriere, France). The FunguyD240 bacterial endotoxin/fungal glucan detector (Dynamiker Biotechnology (Tianjin) Co., Ltd., China) was used to perform the G test. Epstein–Barr virus and human cytomegalovirus were detected using fluorescence quantitative PCR assay kits (Guangzhou Daan Gene Co., LTD., China).

### Definition of sensitivity, specificity, and predictive values

The sensitivity, specificity, positive predictive value (PPV), and negative predictive value (NPV) were defined as previously reported.^17^ Sensitivity = (positive samples in BSI group)/(samples in BSI group) × 100%, specificity = (negative samples in non-BSI group)/(samples in non-BSI group) × 100%, positive predictive value = (positive samples in BSI group)/[(positive samples in BSI group) + (positive detection in non-BSI group)] × 100%, negative predictive value = (negative samples in non-BSI group)/[(negative samples in non-BSI group) + (negative samples in BSI group)] × 100%.

### Statistical analysis

Comparative analysis was conducted by Chi-square test, Fisher exact test, or the McNemar test for discrete variables, as appropriate. The sensitivity, specificity, PPV, and NPV of mNGS and reference tests, as well as their corresponding 95% confidence intervals (CIs) were calculated by exact (Clopper–Pearson) methods. Analysis of correlation between read abundance and clinical diagnosis was conducted by Kendall correlation test. Data analyses were performed by using SPSS 26.0 software, and *P* values < 0.05 were considered statistically significant.

## Results

### Characteristics of the patients and the samples

The demographic information and clinical characteristics of the patients and samples were shown in Table 1. A total of 224 samples were included, 223 of which underwent mNGS (metaDNA-seq), 1 underwent metatranscriptomic sequencing (metaRNA-seq), and 8 were submitted for both metaDNA-seq and metaRNA-seq.

**Table 1:** Demographic information and clinical characteristics of the patients and samples.

Characteristics	Samples^a^ (*n* = 224)
Age, months	89.9 (43.7, 152.0)^b^
0–12	15 (6.7%)
12–36	31 (13.8%)
36–60	28 (12.5%)
>60	150 (70.0%)
Sex	
Male	143 (63.8%)
Female	81 (36.2%)
Hematological malignancies	198 (88.4%)
ALL	96 (42.9%)
AML	48 (21.4%)
NHL	46 (20.5%)
MAL	4 (1.8%)
JMML	3 (1.3%)
MPN	1 (0.4%)
Solid tumors	26 (11.6%)
NB	13 (5.8%)
HB	4 (1.8%)
RMS	3 (1.3%)
GNB	2 (0.9%)
Others^c^	4 (0.9%)
Department	
PICU	134 (59.8%)
Hematology	90 (40.2%)
Absolute neutrophil counts (ANC)	141
<0.5 × 10^9^/L	73 (51.8%)
≥0.5 × 10^9^/L	68 (48.2%)

*ALL* acute lymphoblastic leukemia, *AML* acute myeloid leukemia, *NHL* non-Hodgkin’s lymphoma, *MAL* mixed acute leukemia, *JMML* juvenile myelomonocytic leukemia, *MPN* myeloproliferative neoplasm, *NB* neuroblastoma, *HB* hepatoblastoma, *RMS* rhabdomyosarcoma, *PB* pineoblastomas, *PICU* pediatric intensive care unit.

^a^Data are given as *n* (%) unless otherwise specified.

^b^Data are given as median [interquartile range (IQR: 25th–75th percentiles)].

^c^Others includes 1 with intravascular angiomatosis, 1 with thoracic lymphangioma, 1 with Wilms’ tumor, and 1 with pleuropulmonary blastoma.

### Results of mNGS and reference tests regardless of clinical relevance

**Fig. 2:**
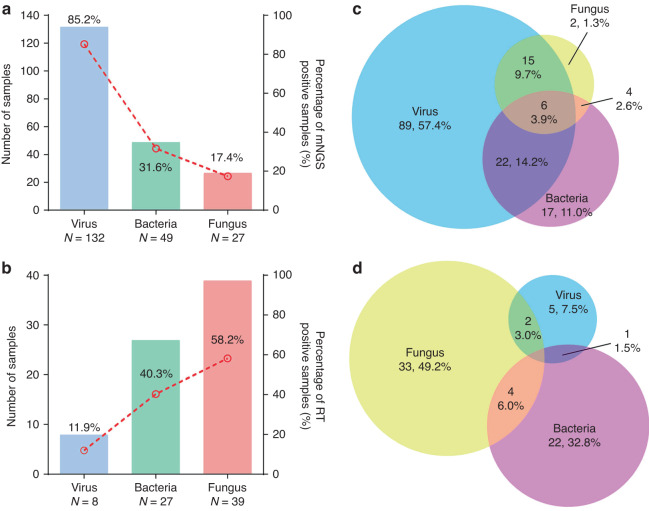
Results of mNGS and reference tests. **a**, **b** Classification of pathogens detected by mNGS and RT. **c**, **d** Venn diagram showing the overlap of virus, bacteria and fungus detected by mNGS and RT.

The overall positive detection rate of mNGS regardless of clinical relevance was 69.2% (155/224). Among all the pathogens detected by mNGS, virus was the most commonly detected pathogen category (132/155, 85.2%), followed by bacteria (49/155, 31.6%) and fungus (27/155, 17.4%) (Fig. 2a). In contrast, reference tests only provide positive results in 29.9% of the samples (67/224), significantly lower than the mNGS results as expected (*P* < 0.001). Fungus was the most commonly detected pathogen category (39/67, 58.2%), followed by bacteria (27/67, 40.3%), and virus (8/67, 11.9%) in reference tests, showing a different pattern from mNGS (Fig. 2b).

Among all the 155 mNGS-positive samples, 47 samples (30.3%) were detected with more than one pathogen category (Fig. 2c), with bacteria-virus co-detection (28/47, 59.6%) being the most common type. Reference tests detected more than one pathogen category in 10.4% (7 samples) in all the 67 positive samples (Fig. 2d), which was also significantly lower than that of mNGS (*P* < 0.001). Such apparent difference is mainly due to the limited ability in detecting virus of the reference tests (132 vs 8 virus-positive samples, *P* < 0.001).

### Comparison of the results between mNGS and reference tests

**Fig. 3:**
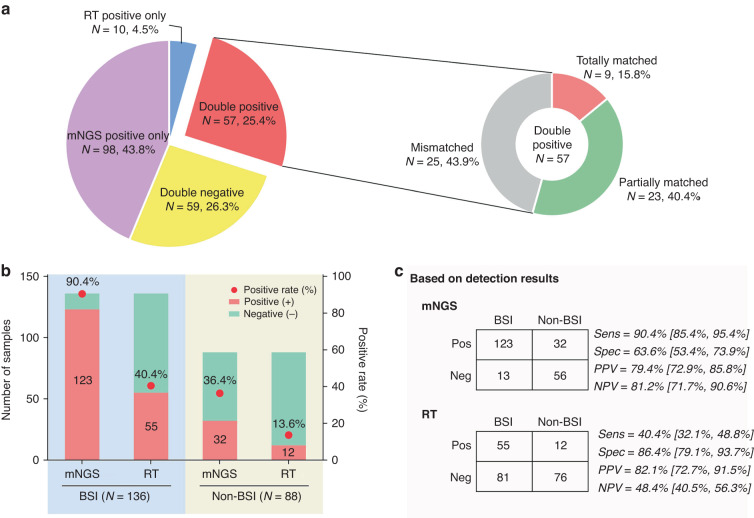
Comparation of the results from mNGS and reference tests. **a** Concordance analysis of the results from mNGS and RT. Pie chart showing the numbers and proportions of double positive, double negative and single positive of mNGS and reference tests. Double positive samples were further divided into totally matched, partially matched (at least one same detected pathogen between the two tests) and mismatched group, and their numbers and proportions were shown in the sub-pie chart. **b** Comparation of the positive detection rate between mNGS and RT in samples with bloodstream infection (BIS) and non-BIS. **c** Comparation of the performance of mNGS from RT were shown, including sensitivity, specificity, positive predictive value (PPV), and negative predictive value (NPV). Sens sensitivity, Spec specificity.

To compare the concordance between mNGS and reference tests, results from the two tests were divided into four groups (Fig. 3a). Fifty-seven samples (25.4%) were positive for both methods, while 59 samples (26.3%) were both negative. Further analysis of the double positive samples showed that the pathogens detected by the two methods were totally matched in only 15.8% samples (9/57), and were partially matched and completely different in 40.4% (23/57) and 43.9% samples (25/57), respectively. The major reason contributing to the complete difference between the two methods is that 84.0% of the samples (21/25) were diagnosed as fungus positive according to G/GM tests, while mNGS detected bacteria and/or virus in these samples.

According to the final diagnosis determined by two senior clinicians, all the 224 samples were divided into two groups from patients either with BSI or not. In both BSI and non-BSI groups, the positive detection rates from mNGS were significantly higher than that in the reference tests (90.4% vs 40.4%, *P* < 0.001, and 36.4% vs 13.6%, *P* < 0.001, respectively).(Fig. 3c). The NPV of mNGS is about 32.8% higher than that of the reference tests (81.2% vs 48.4%, *P* < 0.001), indicating mNGS is a more reliable method to exclude infection.

### Comparison of clinical diagnostic performance between mNGS and reference tests

According to the final diagnosis, all the 224 results detected by both methods were classified into six categories, (1) proved pathogens in BSI group that were considered as the causative pathogens by the doctors (true positive); (2) uncertain pathogens in BSI group that cannot be excluded as the causative pathogens; (3) unsupported pathogens in BSI group that were not consistent with clinical conditions (false positive); (4) no pathogen being detected in BSI group (false negative); (5) unsupported pathogens in non-BSI group (false positive); and (6) no pathogen being detected in non-BSI group (true negative).

**Fig. 4:**
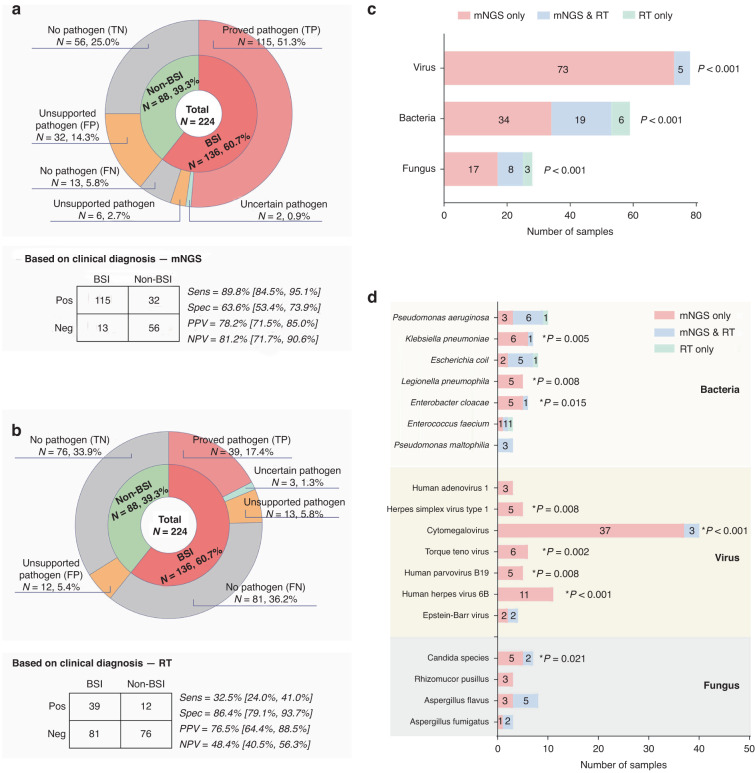
Diagnostic performance of mNGS and reference tests for pathogen detection. **a**, **b** According to the clinical diagnosis, the results of mNGS and RT from BSI samples were classified into proved pathogen, uncertain pathogen, unsupported pathogen and no pathogen, and the results of mNGS and RT from non-BSI samples were classified into unsupported pathogen and no pathogen. Comparation of the diagnostic performance of mNGS from RT were shown, including sensitivity, specificity, PPV, and NPV. **c** Clinical diagnosed pathogens detected by mNGS and RT were shown, and the numbers of viral pathogens, bacterial pathogens, and fungal pathogens detected by mNGS alone, RT alone, or both of the tests according to the clinical diagnosis were shown. **d** Eighteen pathogens detected in ≥3 samples, including 7 bacteria, 7 viruses, and 4 fungus, were listed in the histogram. Sens sensitivity, Spec specificity, PPV positive predictive value, NPV negative predictive value.

mNGS provided significantly more proved pathogens in BSI group than reference tests (84.6%, 115/136 vs 28.7%, 39/136, *P* < 0.001). Consistently, in non-BSI groups, mNGS also provided more false positive results (36.4%, 32/88 vs 13.6%, 12/88, *P* < 0.001) and less true negative results (63.6%, 56/88 vs 86.4%, 76/88, *P* < 0.001) compared to reference tests (Fig. 4a, b). Therefore, mNGS has a much better prediction value for non-BSI in these pediatric oncology patients due to its high sensitivity. Among all the clinician-confirmed pathogens, 94.5% (156/165) can be detected by mNGS, covering 100%, 89.8% and 89.3% of the clinical confirmed viral pathogens, bacterial pathogens, and fungal pathogens, respectively. Most importantly, 75.2% of the confirmed pathogen was only detected by mNGS (Fig. 4c). Eighteen species of pathogens have been confirmed as causative pathogens in ≥3 samples. mNGS detected 97.8% (134/137) of these pathogens, showing apparent advantage over reference tests (24.8%, 34/137, *P* < 0.001), especially for virus (Fig. 4d).

Out of the 9 samples that were examined using RNA seq, 3 were diagnosed with BSI, and 2 of those samples were identified with clinically recognized viruses, including CMV and HHV6. The detailed information of these 9 samples was listed in Supplementary Table 2. Correlation between the results of reference tests for NGS-positive samples for all the clinician-confirmed pathogens was shown in Supplementary Table 3.

### Interpretation of the mNGS results

**Fig. 5:**
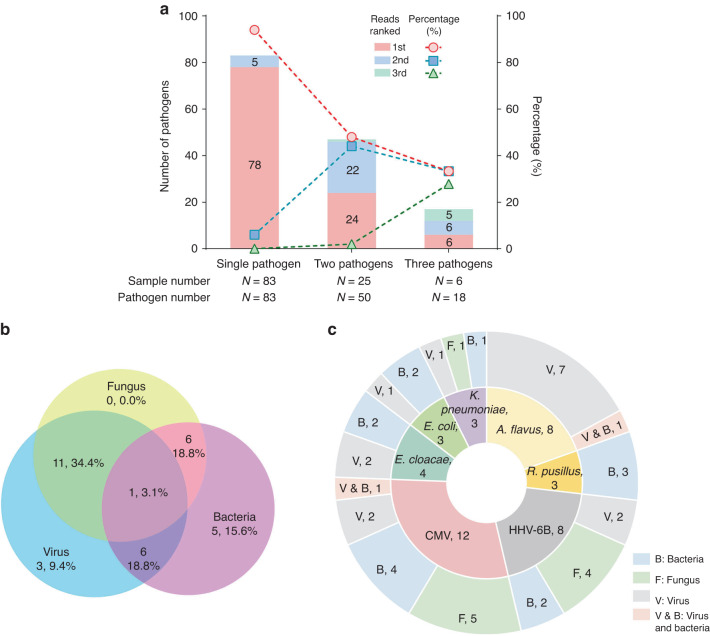
Types of clinical diagnosed pathogens detected by mNGS. **a** Correlation between clinical diagnosed pathogens with mNGS sequencing read abundance. **b** Venn diagram showing the overlap of mixed pathogens infections detected by mNGS (pathogens ≥2 in each sample). **c** Microorganisms that were susceptible to co-infected with other organisms were shown (episodes of co-infection ≥3 samples).

The detection of multiple pathogens in one sample is of a great challenge for clinical interpretation of mNGS results. Therefore, we analyzed the characteristics of the clinical confirmed pathogens in all the 115 “true positive” samples. Eighty-three samples showed single-pathogen infection, and the causative pathogen was the most abundant one in 94.0% of the samples (Fig. 5a). Consistent with the single-pathogen infection samples, among the samples with dual and triple causative pathogens, the pathogens with the most abundant reads were diagnosed as the causative pathogens in 92.0% and 94.4% samples, respectively. There was a significant correlation between read abundance and the clinical diagnosis (Kendall’s tau *b* = 0.752, *P* < 0.001).

In the 32 multiple-pathogen infected samples, dual-infections with virus and fungus were the most common type in the present study (34.4%, 11/32), followed by bacteria plus virus (18.8%), and bacteria and fungus (18.8%) (Fig. 5b). For the co-infection detected by mNGS, CMV was most susceptible to be co-infected with other microorganisms, followed by HHV-6B, *Aspergillus flavus*, *Enterobacter cloacae*, *Rhizomucor pusillus*, *Escherichia coli*, and *Klebsiella pneumoniae* (Fig. 5c). In contrast, reference tests only detected co-infection in 2 samples, one with two species of bacteria, and the other one with bacteria plus virus. Therefore, mNGS can provide valuable information for co-infection diagnosis.

### Effects of neutropenia on the infected pathogen spectrum

Neutropenia is common side effect of chemotherapy in patients with hematological cancer and solid tumor, and has been well demonstrated to increase the risk for infections. To evaluate the impact of neutropenia on the change of pathogen spectrum, BSI samples were divided into 2 groups according to the available absolute neutrophil counts (ANC) of these patients, including neutropenia (ANC < 0.5 × 10^9^/L, *N* = 50) and normal neutrophil counts (ANC ≥ 0.5 × 10^9^/L, *N* = 33).

**Fig. 6:**
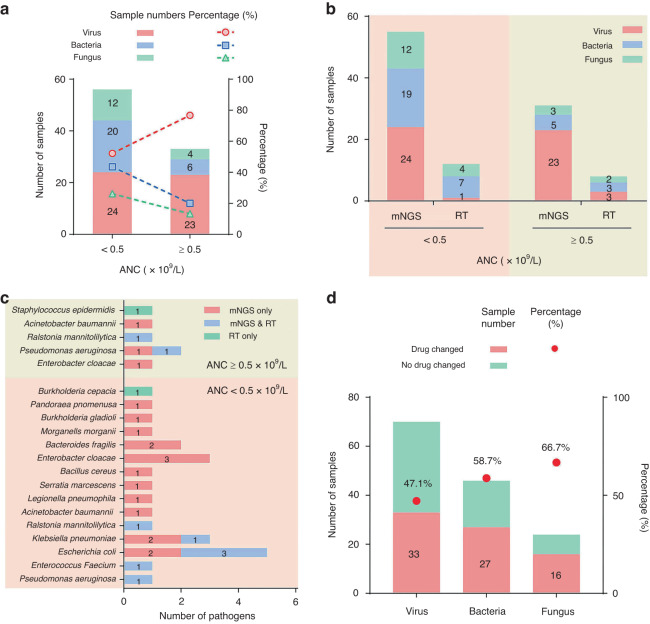
Distribution of clinical diagnosed pathogens in samples with different absolute neutrophil counts (ANC). **a** Samples were divided into 2 groups according to the available absolute neutrophil counts (ANC) of the patients, including neutropenia (ANC < 0.5 × 10^9^) and normal neutrophil counts (ANC ≥ 0.5 × 10^9^), and the distribution of clinical diagnosed pathogens in samples between groups were shown. **b** Types of clinical diagnosed pathogens detected by mNGS and RT in the two ANC groups. **c** The numbers of bacterial pathogens detected by mNGS tests in different ANC groups were shown. **d** A histogram showing the distribution of 40 samples which were not given a previous empirical antiviral treatment in different ANC groups. The proportion of samples which were added antiviral drugs after mNGS tests in these samples was shown.

ANC had no significant effects on the detection rates of mNGS (*P* = 0.520) or reference tests (*P* = 0.791) (Supplementary Fig. 1A, B), but significantly changed the clinical confirmed pathogen spectrum (*P* = 0.049). The neutropenia group had a significantly higher incidence of bacterial infections (43.5% vs 20.0%, *P* = 0.035), but a lower incidence of viral infections (52.2% vs 76.7%, *P* = 0.032) compared to the group with normal ANC (Fig. 6a). No significant difference in the prevalence of fungal infections was observed between the two groups (*P* = 0.183). In both groups, all of the clinical confirmed pathogens could be detected by mNGS, while only very a few by reference tests (Fig. 6b). Like ANC, absolute lymphocyte counts (ALC) had neither significant effect on the detection rates of mNGS (*P* = 0.200) nor reference tests (*P* = 0.200) (Supplementary Fig. 2A, B). Besides, ALC had no significant effect on the clinical confirmed pathogen spectrum (*P* = 0.155) (Supplementary Fig. 2C, D).

Further analysis of clinical confirmed pathogens showed that the most frequently identified bacteria in the present study were *Escherichia coli*, followed by *Enterobacter cloacae*, *Pseudomonas aeruginosa*, and *Klebsiella pneumoniae* (Fig. 6c). The overall infection caused by gram-negative bacteria were significantly more prevalent than those by gram-positive ones (90%, 27/30 vs 10%, 3/30, *P* < 0.001), and most of the identified bacteria were opportunistic bacteria (87.5%, 14/16). Majority of the bacteria were only identified in the neutropenia group (68.8%, 11/16, Fig. 6c). ANC also affected the causative pathogen spectrum of fungus and virus, with the fungus only identified in the neutropenia group were *Rhizomucor pusillus*, *Trichosporon asahii*, *Aspergillus flavus*, and Herpes simplex virus-1 (HSV-1) being only detected in the neutropenia group (Supplementary Fig. 1C, D).

### Impacts of mNGS results on clinical outcome

To determine the impact of mNGS results on clinical treatment, we analyzed the usage of antimicrobial regimens in the 115 true-positive samples with proved pathogens detected by mNGS. Among the 46 episodes with clinical confirmed bacterial infection, 27 (58.7%) changed antibacterial drug regimens after mNGS pathogen detection, including antibiotics escalation in 25 cases and de-escalation in 2 cases. Among the 70 episodes of clinical confirmed viruses and 24 episodes of clinical confirmed fungus, addition of antiviral or antifungal drugs were observed in 33 (47.1%) and 16 (66.7%) cases, respectively (Fig. 6d). Therefore, mNGS results had an impact on the antimicrobial regimens’ usage in more than half of the samples (54.3%, 76/140) in the present study.

Regarding the modification of antibiotic regimens in 27 cases, 8 cases (29.6%) experienced relief after upgrading their antibiotic regimen. For the 16 cases that added antifungal drugs, 2 cases (12.5%) experienced relief. In the 33 cases that added antiviral drugs, the majority of (22 case, 66.7%) cases experienced relief. Overall, there were 42.1% (32/76) patients showed relief after changing treatment based on mNGS results.

## Discussion

Prompt and accurate identification of causative pathogens can help precise treatment and improve the BSI patients’ outcome. Herein, we evaluated the diagnostic performance of mNGS and its clinical impact in pediatric oncology patients suspected with BSI. Consistently with previous reports,^3,18^ mNGS demonstrated much higher sensitivity compared to reference tests, especially for virus. Among the 136 clinical confirmed BSI samples, mNGS provided pathogen results for 115 samples (84.6%). Only 13 samples (9.6%) were clinical confirmed as “false negative.” Therefore, mNGS provided a much better NPV compared to reference tests, and the clinicians can consider a high probability of non-BSI if the mNGS result is negative.

In the present study, we also observed that the mNGS results were in good agreement (76.3%) with clinical diagnosis, with 51.3% “true positive” and 25.0% “true negative.” This is much higher than the all-sample-type results we reported previously using the same detection platform (47.3% “true positive and 14.4% “true negative”).^17^ The sensitivity of mNGS is similar regardless of clinical relevance (90.4% vs 89.8%). However, when considering clinical relevance, the sensitivity of reference tests significantly decreases (40.4% vs 32.5%, *P* < 0.001). This is because microorganisms detected in 16 samples were not considered to be disease-causing pathogens by clinicians, most of which (14/16, 87.5%) were G/GM test positive. Because a variety of exposure factors, including age, existing disease, infected bacteria, antibiotics, and nutritional support, may influence the results of G/GM test,^19^ mNGS will be a better method for fungus diagnosis.

mNGS has significant advantages in pathogen detection for immunocompromised hosts, making it to be a recommended first-line diagnostic tool or an additional test for reference tests.^20^ In this study, except for CMV and EBV, 93.6% viruses (73/78) can only be detected by mNGS. However, the bacteria and fungi only detected by mNGS was significantly lower than virus, accounting for 57.6% (34/59) and 60.7% (17/28) of all clinically diagnosed pathogens, respectively. The low virus detection rate in reference tests is due to absence of clinical detection methods, whereas the low bacteria and fungi detection rate is primarily due to their challenging culturing process, as well as the use of antibiotics.

mNGS produced some false-positive results in this study. All false positive pathogens in non-BSI group were virus. Of them, TTV was detected in 21 false-positive samples, with lower sequencing reads (<100 in 19 samples). In contrast, true-positive samples had higher sequencing reads (329–1212). Similarly, CMV was detected in 8 false-positive samples with lower sequencing reads (3–14) compared to 44 true-positive samples. BK polyomavirus was detected in 5 false-positive samples with low sequencing reads (2-13). These results indicate that detecting low-abundance DNA viruses was the primary cause of false-positive results in this population. Of note, although mNGS provides a powerful tool for detecting potential pathogens, not all detected microorganisms are diseases-causing pathogens. Additional laboratory tests, such as cultures or serology, and clinical judgment from highly qualified infectious disease experts are highly needed to rule out false positives.

In this study, we observed ten samples that were positive by reference tests but negative by mNGS. All five non-BSI samples were G/GM positive, while the five BSI samples included two G-positive and three blood culture-positive samples. The three blood culture-positive bacteria are *Pseudomonas aeruginosa*, *Burkholderia cepacia*, and *Streptococcus bradygii*, respectively, which are often associated with BSI, especially in immunocompromised patients. Detecting fungal infections using mNGS is more challenging due to difficulties in lysing their cell walls, leading to their lower sensitivity. Therefore, despite the high rate of false positives in G/GM tests, their results should still be considered. Hence, apart from pathogen identification, accurate diagnosis of BSI requires a comprehensive analysis of the patient’s medical history, clinical symptoms, and imaging examinations, especially for samples that positive for G/GM test. In fact, when employing mNGS for microbial plasma cell-free DNA (mcfDNA) detection, different pathogens demonstrate varying detection limits, ranging from 10 to 1000 copies/ml.^21^ Below detection limits, mcfDNA could not be detected, but conventional culture amplifies low pathogen counts, further facilitating the detection. Therefore, for suspected bacterial or fungal infections, performing both mNGS and reference tests is advisable.

Severe neutropenia is a common side effect caused by chemotherapy in oncology patients, increases the risk of bacterial infections.^22^ Consistent with previous reports,^23,24^ we observed significantly more infection caused by bacteria, but not by virus or fungus, in patients with neutropenia. Most of the identified bacteria were opportunistic bacteria. More importantly, 90% of the bacterial infections were caused by gram-negative bacteria. *Escherichia coli*, *Enterobacter cloacae*, *Klebsiella pneumoniae, Pseudomonas aeruginosa* were the most common pathogens, accounting for 50% of the total bacterial infections.. This result indicated that when the pathogen is unknown, the empirical use of drugs against gram-negative bacteria should be considered first in our center.

Our study also have some limitations. Firstly, mNGS was performed on the majority of samples collected on different days than the day of reference tests in the present study, which could potentially lead to inconsistencies between the results of mNGS and reference tests. Secondly, as a one-arm real-world study, evaluating the impact of mNGS on BSI is challenging because of lacking control group. Future case-control studies or randomized controlled trials are still needed to evaluate the impact of mNGS on BSI.

In summary, our results demonstrated that mNGS was effective for pathogen diagnosis in pediatric oncology patients suspected with BSI, especially for virus. BSI caused by Gram-negative bacteria was more prevalent in the present study, and neutropenia is a risk factor for susceptibility to bacterial infections in pediatric oncology patients. Prompt identification of causative microorganism through mNGS can help clinicians to adjust antimicrobial drug regimens in time, which might further improve the outcome of BSI.

### Supplementary information

Figure S1

Figure S2

Supplementary Figure legends

Supplementary Table 1

Supplementary Table 2

Supplementary Table 3

## Data Availability

The data that support the finding of this study are available from the corresponding author upon reasonable request.
