# Supplementary figures and images for: Metagenomic next-generation sequencing in detecting pathogens in pediatric oncology patients with suspected bloodstream infections
Source: Pediatr Res. 2023 Oct 19;95(3):843–51. doi: 10.1038/s41390-023-02776-y (PMC10899103; doi:10.1038/s41390-023-02776-y)

Figure S1

A

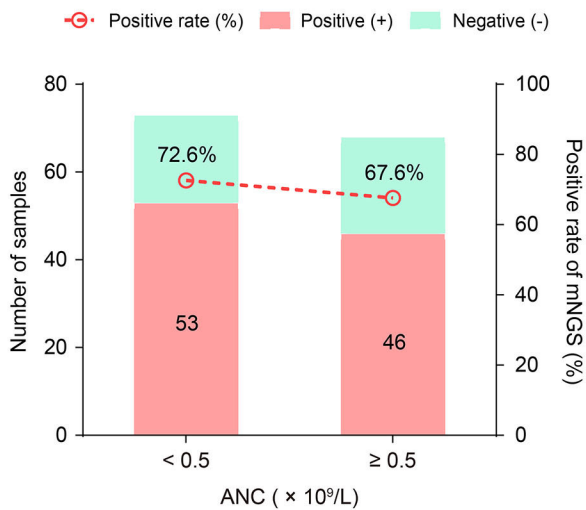

B

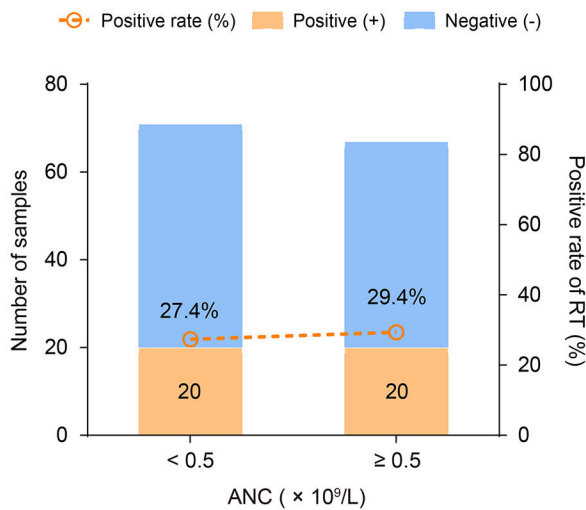

C

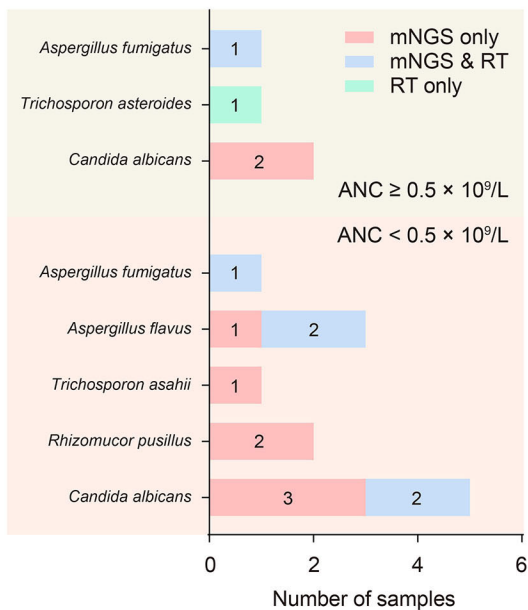

D

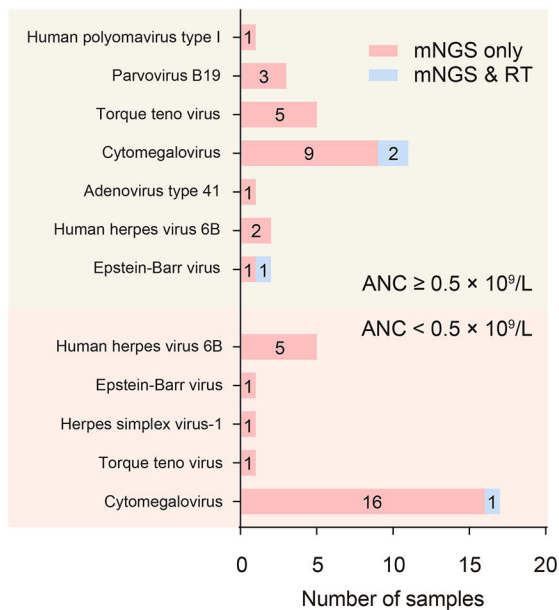

Supplement: Supplementary file 1 — Figure S1 [file 41390_2023_2776_MOESM1_ESM.pdf]

Figure S2

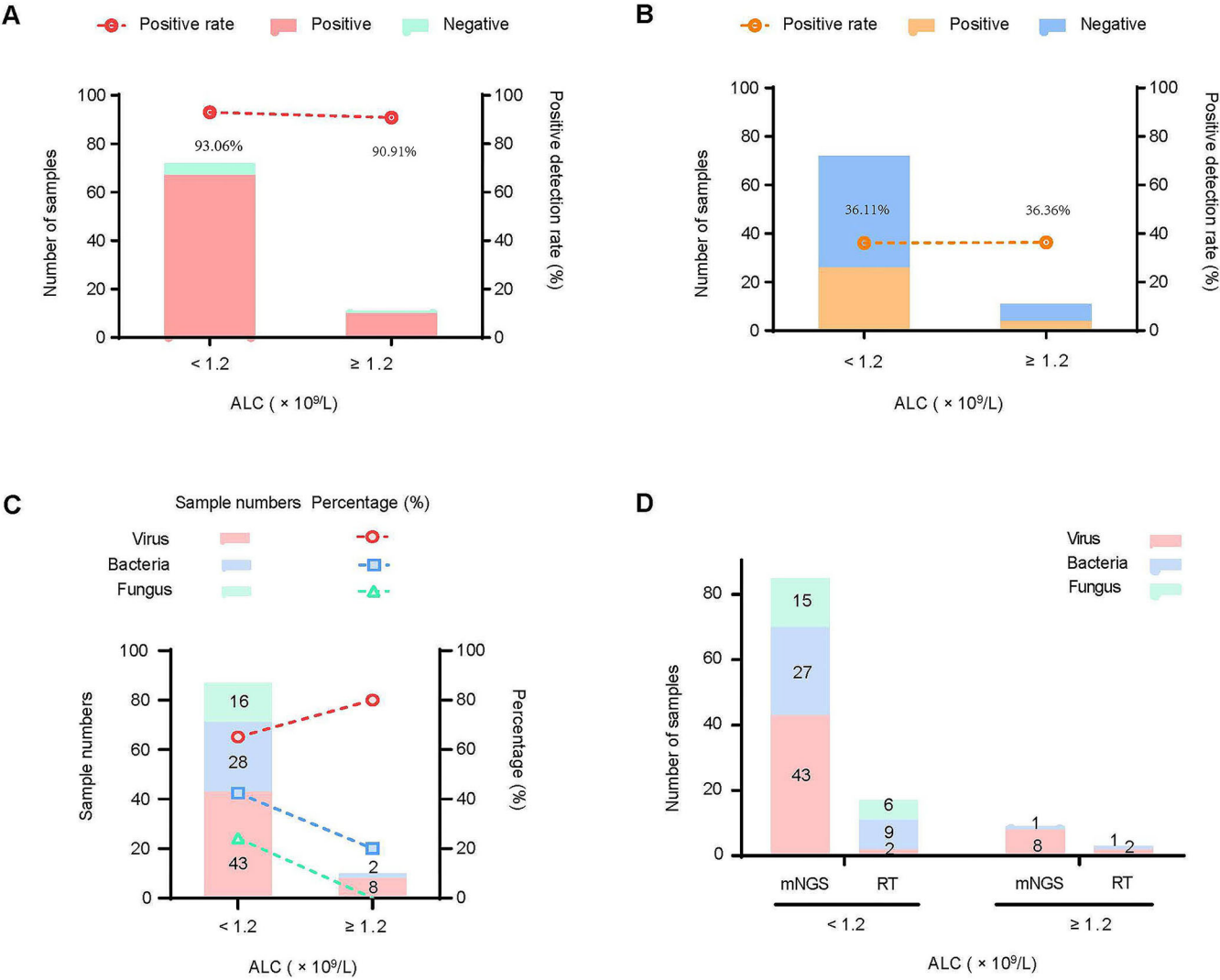

Supplement: Supplementary file 2 — Figure S2 [file 41390_2023_2776_MOESM2_ESM.pdf]
