# Supplementary material for: Metagenomic next-generation sequencing in detecting pathogens in pediatric oncology patients with suspected bloodstream infections
Source: Pediatr Res. 2023 Oct 19;95(3):843–51. doi: 10.1038/s41390-023-02776-y (PMC10899103; doi:10.1038/s41390-023-02776-y)
Supplement: Supplementary file 3 — Supplementary Figure legends [file 41390_2023_2776_MOESM3_ESM.pdf]

Supplementary Figure legends

Supplementary Figure 1. Distribution of clinical diagnosed fungus and virus in samples with different absolute neutrophil counts (ANC). (A and B) The overall positive detection rate of mNGS and RT in neutropenia (ANC$<0.5\times10^9$) and normal neutrophil counts (ANC$\geq0.5\times10^9$) groups. (C and D) The numbers of fungal and viral pathogens detected by mNGS tests in different ANC groups were shown.

Supplementary Figure 2. Distribution of clinical diagnosed fungus and virus in samples with different absolute lymphocyte counts (ALC). Samples were divided into 2 groups according to the available absolute lymphocyte counts (ALC) of the patients, including low ALC (ANC$<1.2\times10^9$) and normal ALC (ANC$\geq1.2\times10^9$) groups. (A and B) The overall positive detection rate of mNGS and RT in low ALC and normal ALC groups. (C) The distribution of clinical diagnosed pathogens in samples between groups were shown. B. Types of clinical diagnosed pathogens detected by mNGS and RT in the two ALC groups
