# Supplementary material for: Metagenomic next-generation sequencing in detecting pathogens in pediatric oncology patients with suspected bloodstream infections
Source: Pediatr Res. 2023 Oct 19;95(3):843–51. doi: 10.1038/s41390-023-02776-y (PMC10899103; doi:10.1038/s41390-023-02776-y)
Supplement: Supplementary file 5 — Supplementary Table 2 [file 41390_2023_2776_MOESM5_ESM.pdf]

Supplementary Table 2. Pathogens detected using metaRNA-seq alone or a combination of metaRNA-seq & metaDNA-seq.

No.	Group	Type of mNGS	Pathogens detected by metaDNA-seq	Pathogens detected by metaRNA-seq	Pathogens detected by RT	Clinical confirmed pathogens
1	BSI	metaRNA-seq	-	CMV	-	CMV
2	BSI	metaDNA-seq & metaRNA-seq	-	HHV6 and TTV	-	HHV6
3	BSI	metaDNA-seq & metaRNA-seq	CMV, HSV1 and Adenovirus	-	-	-
4	Non-BSI	metaDNA-seq & metaRNA-seq	-	-	-	-
5	Non-BSI	metaDNA-seq & metaRNA-seq	-	-	-	-
6	Non-BSI	metaDNA-seq & metaRNA-seq	CMV	-	-	-
7	Non-BSI	metaDNA-seq & metaRNA-seq	TTV	TTV and Bocavirus	GM test positive	-
8	Non-BSI	metaDNA-seq & metaRNA-seq	-	-	-	-
9	Non-BSI	metaDNA-seq & metaRNA-seq	-	-	-	-

RT: reference test, BSI: bloodstream infections, CMV: Cytomegalovirus, HSV1: Herpes simplex virus type 1, TTV: Torque teno virus, GM: Galactomannan
